# Determinants of Metabolic Syndrome in Virally Suppressed People Living with HIV: A Single-Center Comparative Analysis of Antiretroviral Regimens

**DOI:** 10.3390/metabo16090631

**Published:** 2026-08-29

**Authors:** Michał Biały, Małgorzata Inglot

**Affiliations:** Department of Infectious Diseases, Liver Diseases and Acquired Immune Deficiencies, Wroclaw Medical University, 51-149 Wroclaw, Poland

**Keywords:** HIV, antiretroviral therapy (ART), metabolic syndrome (MetS), weight gain, integrase strand transfer inhibitors (INSTI), tenofovir alafenamide (TAF)

## Abstract

**Background:** Metabolic complications and weight gain have emerged as primary concerns among virologically suppressed and immunologically competent people living with HIV. Particularly integrase strand transfer inhibitors (INSTIs) and tenofovir alafenamide (TAF) are being connected with this issue. **Objectives:** This study aimed to evaluate the prevalence of metabolic syndrome (MetS) and its components within a highly homogeneous, virologically suppressed cohort, assessing the impact of antiretroviral therapy (ART) and HIV-unrelated factors. **Methods:** We conducted a single-center cross-sectional analysis of 93 HIV-positive patients receiving stable ART for >2 years, in Wrocław, Poland. To minimize confounding, individuals with HBV/HCV co-infections or significant alcohol use were excluded. Participants were stratified primarily by regimen, INSTI/TAF-based (*n* = 50) vs. PI/TAF-based (*n* = 28), and secondarily by the particular drugs taken. Clinical assessment included anthropometric parameters, lipid profiles, and glucose metabolism. **Results:** The prevalence of MetS was 10.8%. No statistically significant differences were observed in the incidence of MetS, insulin resistance, or dyslipidemia between the INSTI/TAF and PI/TAF groups. A marginally significant trend toward lower total cholesterol was identified in patients receiving dolutegravir/TAF compared to those on darunavir/TAF. **Conclusions:** In this stable Caucasian cohort with long-term viral suppression, we found no statistically significant differences in metabolic outcomes between modern INSTI- and PI-based regimens. However, due to the limited sample size and low statistical power, these findings do not prove clinical equivalence. Our results suggest that in particular tightly selected settings of well-controlled patients, traditional risk factors and lifestyle may remain the primary drivers of metabolic health, although modest drug-related effects cannot be ruled out.

## 1. Introduction and Study Aim

### 1.1. Metabolic Disorders Among People Living with HIV

With life expectancy of people living with HIV being comparable to the general population [1], serious non-AIDS events (SNAEs), including cardiometabolic complications, have surpassed AIDS-defining diseases as the major source of morbidity and mortality in people living with HIV. Therefore, clinical attention in this group of patients has redirected towards managing metabolic (and cardiovascular) disorders, as they are the primary health concern in virologically suppressed populations [2].

The weight recovery is usually expected following the initiation of antiretroviral therapy (ART). However, recent reports suggest that modern regimens (albeit being much safer than previous generations), particularly those containing integrase strand transfer inhibitors (INSTIs) and tenofovir alafenamide (TAF), may independently drive excessive weight gain and metabolic burden [3,4,5].

Large observational cohorts and trial emulations have recently linked INSTI and TAF use to an increased risk of incident hypertension, dyslipidemia, and diabetes. For instance, data from the REPRIEVE trial emulation over a 5-year follow-up demonstrated an elevated risk of obesity, diabetes, and hypertension following a switch to an INSTI-based regimen [6]. Similarly, the RESPOND consortium highlighted that INSTI use is associated with incident hypertension, while TAF use correlates with dyslipidemia, with both pathways appearing to be partially mediated by weight gain [5]. Furthermore, contemporary updates emphasize that changing health-related behaviors and the increasing burden of medications used to treat comorbidities further complicate the clinical picture in aging people living with HIV.

Despite these findings, it is difficult to isolate drug-specific metabolic effects from the broader “return to health” effect or individual lifestyle changes [7]. Also, data from real-world settings are often confounded by socioeconomic instability, disparate access to healthcare, or co-infections such as viral hepatitis [8].

### 1.2. Rationale and Study Aim

Given the heterogeneous nature of large, multicenter cohorts, there is an essential need for studies evaluating metabolic health in highly controlled, homogenous populations. The primary rationale for this study stems from the need to minimize major confounders—such as hepatitis viruses co-infections, advanced uncompensated comorbidities, and severe chronic liver diseases—which often surpass the direct metabolic impact of contemporary ART regimens in larger datasets.

The aim of this study was to evaluate the prevalence of metabolic syndrome (MetS) and its components in a tightly selected, homogeneous cohort of virally suppressed people living with HIV. By focusing on a uniform single-center population, we aimed to determine whether specific ART classes (INSTI vs. PI) or backbones (TAF vs. others) independently drive metabolic burden, or if the non-HIV-related factors and traditional lifestyle risks remain the primary determinants of metabolic health in this specific setting.

## 2. Material and Methods

### 2.1. Study Design

We performed a cross-sectional observational study of adult people living with HIV treated with ART. Recruitment took place between April 2021 and October 2021. The cohort represents HIV-positive patients living in an urban population in a large (over 800,000 inhabitants) and well-developed metropolitan area with guaranteed access to publicly funded, modern antiretroviral therapy and regular medical monitoring.

This analysis is part of a larger ongoing project evaluating metabolic and hepatic health in people living with HIV, which includes comparisons with healthy controls. Related findings, specifically to metabolic dysfunction-associated steatotic liver disease (MASLD), are reported separately. The methodology of recruitment and data collection is consistent with these associated studies.

Participants were recruited from the Outpatient Clinic for Acquired Immunodeficiency in Wrocław. Inclusion criteria were confirmed HIV infection, stable antiretroviral therapy (ART) for at least two years, age 18–65 years, and absence of hepatitis B (HBV) or hepatitis C (HCV) co-infection.

To maintain cohort homogeneity and minimize impact of confounding factors, we applied strict exclusion criteria. These were: (1) uncontrolled endocrine disorders (e.g., thyroid, parathyroid, or adrenal dysfunction); (2) any chronic liver disease other than MASLD (including alcoholic liver disease, autoimmune hepatitis, primary sclerosing cholangitis primary biliary cholangitis drug-induced liver injury, hemochromatosis, Wilson disease, or α-1-antitrypsin deficiency); (3) severe liver complications (decompensation, encephalopathy, ascites, variceal bleeding, or hepatocellular carcinoma); (4) active malignancy; (5) pregnancy; (6) type 1 diabetes mellitus.

From an initial pool of 2061 patients attending the clinic, 608 were identified as meeting the preliminary inclusion criteria (we noticed a significant exclusion rate at this point mainly due to HBV or HCV co-infection in many of our patients). Subsequently, 216 individuals were further excluded based on the detailed exclusion criteria, with advanced alcoholic liver disease and its complications being the predominant reason. This process left 392 patients eligible for the study. All eligible patients with scheduled appointments at the clinic between April and October 2021 were extended an invitation to participate. Ultimately, 93 individuals were enrolled. Upon enrollment of each patient, biological samples were collected, and a self-reported questionnaire was completed. The low enrollment rate (93 out of 392 eligible patients) was a direct result of the COVID-19 pandemic protocols active during the recruitment period. Our clinic transitioned primarily to teleconsultations. Consequently, physical examinations and blood sampling were only feasible for patients who required an in-person visit.

**Figure 1:**
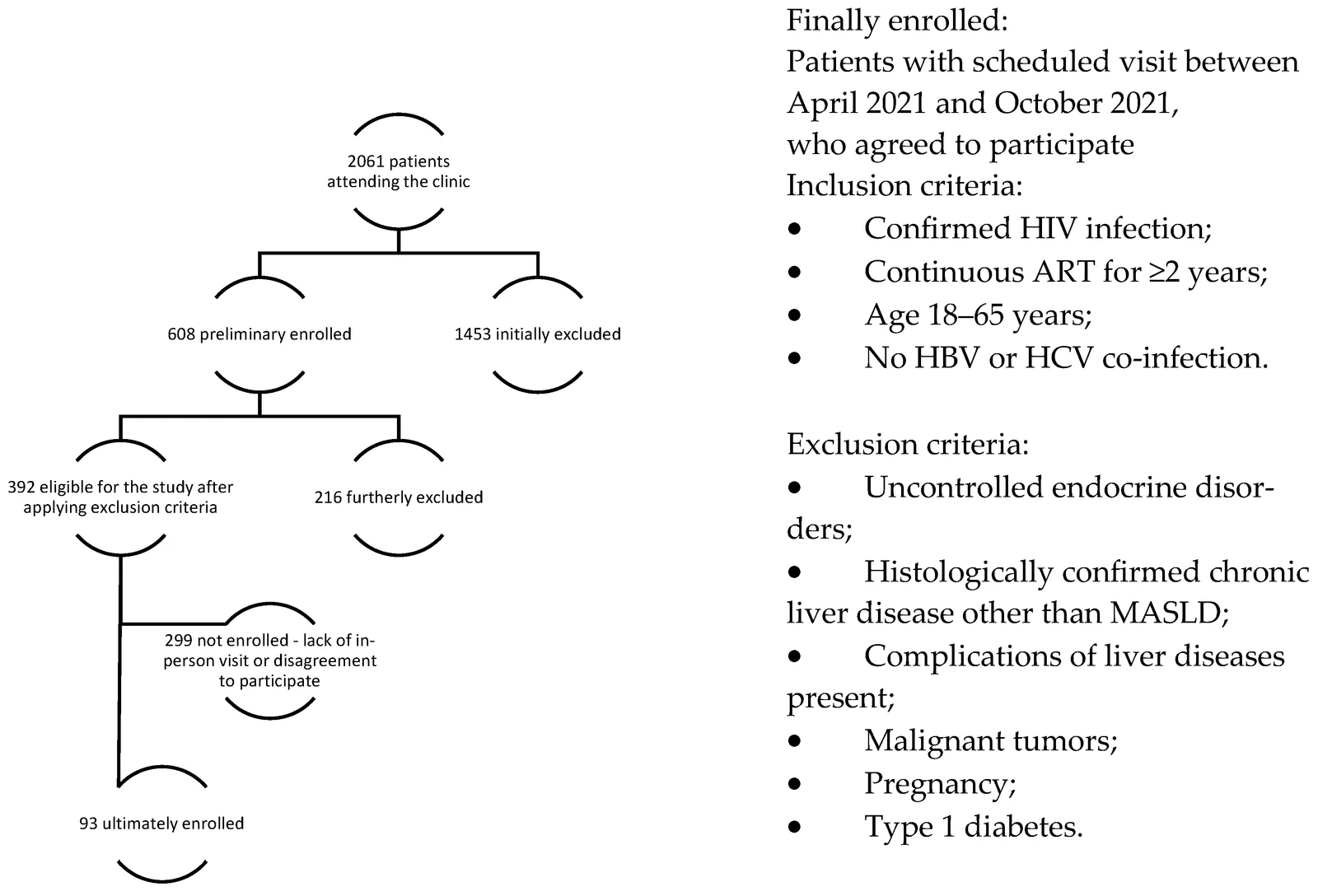
Study group selection.

The pathway of patient selection is presented in Figure 1.

### 2.2. Clinical and Biological Parameters

#### 2.2.1. Data Collection

Data were collected using a structured questionnaire covering demographics (age, sex), anthropometrics (height, current weight, minimum/maximum adult weight), and lifestyle factors (alcohol, tobacco, recreational drug use). Physical activity was evaluated using a custom semi-quantitative scoring system. Coefficients were assigned based on self-reported intensity: 0.25 for light exertion (e.g., slow walking), 0.5 for moderate activity (e.g., brisk walking), 1.0 for vigorous exercise (e.g., jogging, gym workouts), and 1.5 for high-intensity efforts (e.g., running, interval training). Alcohol consumption was converted into standard units, where one unit represented 10 g of pure ethanol [9].

The questionnaire used to assess lifestyle variables was an exploratory, custom-designed tool which was not formally validated or pilot-tested. The physical activity scoring system used in this study was arbitrary and semi-quantitative. As the data were self-reported, the results are inherently subject to recall bias.

HIV-related clinical data, including infection duration, nadir CD4 count, and detailed antiretroviral treatment history, were retrieved from medical records.

#### 2.2.2. Laboratory Analysis

Venous blood samples were collected following an 8–12 h fasting period. Laboratory assessments included the following:

- HIV markers: Viral load and CD4+ T-cell count.
- Hematology and Inflammation: Complete blood count (CBC) with differential and C-reactive protein (CRP).
- Lipid profile: Total cholesterol (TC), HDL-cholesterol (HDL-C), non-HDL cholesterol (non-HDL-C), LDL-cholesterol (LDL-C), and triglycerides (TG).
- Carbohydrate metabolism: Fasting glucose, glycated hemoglobin (HbA1c), and insulin.

#### 2.2.3. Analytical Methods

Routine biochemical analyses were performed in an accredited laboratory (by Polish Centre for Accreditation). HIV-related parameters (viral load, CD4) were conducted at the Wrocław Medical University Laboratory.

- HIV viral load was quantified using the Real-Time PCR assay on a cobas^®^ 4800 System (Roche Diagnostics, Mannheim, Germany).
- CD4 cell counts were measured via flow cytometry using a Navios EX (Beckman Coulter, Brea, CA, USA).
- CBC was analyzed on a Sysmex Analyzer (Sysmex Corporation, Kobe, Japan).
- Biochemical parameters (CRP, TC, HDL-C, LDL-C, TG, glucose, insulin) were measured using spectrophotometric and immunoturbidimetric methods on a cobas^®^ analytical unit (Roche Diagnostics, Manheim, Germany).

#### 2.2.4. Calculations

Insulin resistance was assessed using the Homeostatic Model Assessment for Insulin Resistance (HOMA-IR), calculated according to the formula: (fasting insulin [mU/L] × fasting glucose [mmol/L])/22.5 [10].

### 2.3. Statistical Analysis

Quantitative variables are presented as medians with interquartile ranges (shown as Q1–Q3; first quartile; third quartile), while qualitative variables are described as counts (n). Statistical significance was set at *p* < 0.05; values between 0.05 and 0.1 were reported as trends. Group differences were assessed using the Mann–Whitney U test (for two groups), Kruskal–Wallis test (for 3 groups), or Chi-square test (for categorical variables). Correlations between continuous parameters were evaluated using Spearman’s rank correlation coefficient (R). To quantify the magnitude of the observed differences, effect sizes were calculated. The rank-biserial correlation (*r*) was used for continuous variables compared between two groups (Mann–Whitney U test), and eta-squared (η^2^) for multiple groups (Kruskal–Wallis test). For categorical variables, the Odds Ratio (OR) with a 95% Confidence Interval (95% CI) was calculated for two-group comparisons, and Cramer’s V was used for multiple-group comparisons.

A post hoc power analysis was performed to determine the minimum detectable difference (MDD) for key metabolic parameters (total cholesterol and HOMA-IR) between the main comparative groups (INSTI/TAF vs. PI/TAF). Calculations assumed a two-tailed alpha of 0.05 and 80% statistical power. For this approximation, standard deviations were estimated from interquartile ranges.

Analyses were performed using Statistica 13.3 (TIBCO Software Inc., Palo Alto, CA, USA).

### 2.4. Patient Cohort Stratification

Among the 93 participants, varying ART regimens were identified. For comparative analysis, the cohort was stratified as follows:

Main comparative groups:

- Group A: Integrase strand transfer inhibitor + tenofovir alafenamide (INSTI/TAF, *n* = 50).
- Group B: Protease inhibitor + tenofovir alafenamide (PI/TAF, *n* = 28).

Specific regimen subgroups:

- Group C: Elvitegravir/cobicistat/emtricitabine/tenofovir alafenamide (ELV/c/FTC/TAF, *n* = 28); hereinafter “ELV/TAF”.
- Group D: Dolutegravir/emtricitabine/tenofovir alafenamide (DTG/FTC/TAF, *n* = 15); hereinafter “DTG/TAF”.
- Group E: Darunavir/cobicistat/emtricitabine/tenofovir alafenamide (DRV/c/FTC/TAF, *n* = 28); hereinafter “DRV/TAF”.

Of note, Group B (PI/TAF) and Group E (DRV/TAF) comprise the same patients, as all individuals receiving a PI/TAF regimen in this cohort were treated with DRV/c/FTC/TAF.

The remaining 15 participants receiving other regimens (e.g., INSTI/TDF, *n* = 4; PI/TDF, *n* = 1; NNRTI-based, *n* = 8; others, *n* = 2; TDF—tenofovir disoproxil fumarate; NNRTIs—non-nucleoside reverse transcriptase inhibitors) were included in the whole-cohort descriptive analysis but excluded from specific subgroup comparisons due to small sample sizes.

No a priori sample size calculation was performed. The study used a convenience sample restricted by the pandemic timeframe and strict inclusion criteria.

### 2.5. Definitions

MetS was defined according to the 2022 Polish guidelines [11]. The diagnosis required a BMI ≥ 30 kg/m^2^ and at least two of the following: non-HDL-C ≥130 mg/dL, blood pressure ≥130/85 mmHg (or antihypertensive treatment), and fasting glucose ≥100 mg/dL (or diagnosed diabetes).

Additionally, to confirm the consistency of our findings, we performed a sensitivity analysis using the modified National Cholesterol Education Program Adult Treatment Panel III (NCEP ATP III) criteria. Because our methodology strictly operationalized obesity via BMI to eliminate waist circumference measurement bias, we considered BMI ≥ 30 kg/m^2^ as a surrogate for the abdominal obesity criterion. Under these modified ATP III criteria, MetS was defined as the presence of ≥3/5: BMI ≥ 30 kg/m^2^, TG ≥ 150 mg/dL, HDL-C < 40 mg/dL (men) or <50 mg/dL (women), blood pressure ≥ 130/85 mmHg (or specific treatment), and fasting glucose ≥100 mg/dL (or specific treatment).

Low level viremia was considered as <200 copies/mL [12,13].

### 2.6. Use of Large Language Model

Gemini 3 was used for language refinement only.

## 3. Results

### 3.1. Baseline and HIV-Related Characteristics by Regimen

All participants lived in an urban, socioeconomically stable environment. The cohort reflected the epidemiology of HIV in this region: 83% were male, and most infections were sexually acquired, predominantly among men who have sex with men. Healthcare access was uniform. All participants were Caucasian, with comparable distribution of age and sex across groups. Minor and non-specific abnormalities in complete blood counts were observed in only a few patients.

Key demographic and lifestyle parameters showed no major differences between the groups, including BMI, MetS, and lifetime weight amplitude (LWA). MetS was diagnosed in 10 participants (10.8%) according to the 2022 Polish guidelines. In the sensitivity analysis using the modified NCEP ATP III criteria, the overall MetS prevalence was 15.1% (*n* = 14). Consistent with the primary analysis, the modified ATP III MetS incidence did not differ significantly between the INSTI/TAF and PI/TAF groups (*n* = 9 vs. *n* = 4; *p* = 0.67). The comparison of MetS incidence between the INSTI/TAF and PI/TAF groups yielded a modest OR with an exceptionally wide 95% CI, which reflects the low statistical power due to the small number of events and supports the inability to rule out clinically meaningful differences between the regimens. LWA data were accessible for 76 out of 93 participants due to recall problems. Both smoking rates and alcohol consumption were generally low and did not differ significantly between regimens; only a few (four) individuals reported alcohol intake levels surpassing recommended low-risk thresholds [9]. To account for the missing LWA entries, a complete-case sensitivity analysis was performed on the 76 available participants. This analysis confirmed that group comparisons for primary metabolic parameters (including total cholesterol and HOMA-IR) remained consistent with the findings of the full cohort, indicating that the missing data did not distort the main study outcomes.

Regarding current medications, seven participants (7.5%) in the entire cohort were receiving hypolipemic therapy (statins), and eight (8.6%) were treated with antihypertensive drugs. The distribution of these treatments was low and did not differ significantly between the main comparative groups (INSTI/TAF vs. PI/TAF: statins *n* = 4 vs. *n* = 3; antihypertensives *n* = 4 vs. *n* = 3, respectively).

Regarding HIV-specific parameters, the infection was well controlled. Both nadir and current CD4 cell levels were consistent across the various ART regimens. Adherence to ART was universally reported as good by all patients.

While total duration of antiretroviral treatment was similar among groups, patients in the PI/TAF group had been on their current therapy for a significantly longer period compared to the INSTI/TAF group. In the secondary analysis of specific regimens, the ELV/TAF group exhibited a significantly shorter median treatment duration compared to DTG/TAF and DRV/TAF.

Overall, the calculated effect sizes for the observed differences between the ARV regimens were small to negligible. The only moderate effect size was noted for the total duration of cART and the duration of the current regimen, reflecting the longer treatment history in the PI/TAF group.

Detailed characteristics are provided in Table 1.

### 3.2. Metabolic Outcomes

Analysis of lipid and carbohydrate profiles revealed no statistically significant differences between the main comparative groups (INSTI/TAF vs. PI/TAF). In the secondary analysis of specific regimens (ELV/TAF vs. DTG/TAF vs. DRV/TAF), there was a marginally significant trend toward lower TC in the DTG/TAF group compared to the DRV/TAF group. No other statistically significant differences were identified for the remaining lipid parameters or markers of glucose metabolism.

The effect sizes for metabolic parameters and lipid profiles indicated weak or negligible associations, further supporting the lack of substantial clinical differences between the evaluated regimens. Detailed characteristics are provided in Table 2.

A post hoc power calculation revealed that with the current sample sizes for two main compared groups (INSTI/TAF vs. PI/TAF; *n* = 50 vs. *n* = 28), the minimum detectable difference was approximately 33.4 mg/dL for total cholesterol and 1.33 for HOMA-IR. Any true clinical differences smaller than these thresholds could not be reliably detected, confirming the limited statistical power of the comparisons.

### 3.3. Impact of Treatment Duration and Nadir CD4 Count

Nadir CD4 count showed only a singular correlation with metabolic parameters. A weak negative correlation was found between total ART duration and non-HDL cholesterol, accompanied by a similar, negative, non-significant trend for triglycerides.

The duration of the current regimen was not associated with lipid or carbohydrate parameters in the full cohort. An additional sub-analysis restricted to participants receiving either an INSTI or TAF revealed only a marginal, non-significant trend for LDL-C.

Detailed characteristics are provided in Table 3 and Table 4.

## 4. Discussion

In this single-center, cross-sectional analysis of 93 virally suppressed people living with HIV in Poland, we aimed to determine the impact of modern antiretroviral regimens on metabolic health in our patients. We found no statistically significant associations between key HIV-specific factors—namely ART regimen (INSTI/TAF vs. PI/TAF), total ART duration, or nadir CD4 count—and metabolic outcomes, including lipid profile, glycemic control, insulin resistance, or the presence of metabolic syndrome.

The overall prevalence of MetS in our cohort was notably low at 10.8%. In contrast, a recent 2024 global meta-analysis reported a pooled MetS prevalence of 25.6% among ART-treated PLWH, with regional rates reaching up to 30.4% in the Americas [14]. This discrepancy rises from our strict inclusion criteria and different case definitions. We defined MetS using the 2022 Polish guidelines [11], the most current standard during our primary data analysis. These guidelines require obesity as an obligatory criterion and use a unified non-HDL cholesterol threshold instead of separate TG and HDL parameters. To eliminate the inter-operator measurement bias associated with routine outpatient waist circumference assessments, we operationalized the obesity criterion strictly as BMI ≥ 30 kg/m^2^. This acted as a highly restrictive baseline compared to older ATP III (where obesity is not mandatory) or IDF definitions. Consequently, our definition filtered out individuals with central adiposity but a BMI < 30. Combined with the tight exclusion criteria, this approach explains the lower baseline metabolic burden observed in our cohort. Our study took place in 2021, with primary data analysis in 2022–2023, and therefore it predates the current 2026 AHA/ACC Cardiovascular–Kidney–Metabolic (CKM) guidelines, which focus on continuous risk staging.

### 4.1. Comparison with the Recent Literature

Our findings contrast with many recent reports. Large multicenter studies have frequently mentioned INSTI- or TAF-based regimens as independent drivers of metabolic disturbances. Data from the RESPOND cohort (2024) demonstrated a clear connection between INSTI/TAF regimens and BMI gain, dyslipidemia, and hypertension [5]. Similarly, findings presented at CROI 2025 point out an increased rate of metabolic complications (obesity, diabetes, MetS) following the switch to INSTI-based therapies [2]. Furthermore, a recent target trial emulation using data from the global REPRIEVE cohort revealed that switching to an INSTI-containing regimen was associated with an increased risk of incident obesity, diabetes, and hypertension over a 5-year follow-up, although notably without a corresponding increase in major adverse cardiovascular events (MACE) [6].

While our findings diverge from these large-scale studies, the small sample size remains a major limitation. The lack of statistical significance may reflect a Type II error rather than true clinical equivalence; therefore, more subtle metabolic differences could remain undetected. However, the limited N is a direct consequence of our strict exclusion criteria. By excluding patients with HBV/HCV co-infections or chronic liver diseases, we eliminated significant metabolic confounders that often affect the results of larger, more heterogeneous cohorts. In this rigorously selected population, the metabolic impact of modern ARV regimens appears to be less pronounced. Still, as highlighted in several other reports, long-term data through 144 weeks have shown that initial weight increases often reach a plateau, suggesting that early gains may reflect a “return to health” phenomenon rather than ongoing drug adverse metabolic effect [15]. Additionally, another study suggests “weight gain” observed upon switching to INSTI/TAF to be more a result of withdrawing weight-suppressive drugs (such as TDF or efavirenz) than a direct lipogenic effect of the new drugs [16].

A recent review noted that the metabolic effects of INSTI and TAF are often additive with low CD4+ counts and high viral loads, and are modulated by racial and sex-based differences [17]. Our cohort comprised predominantly male, Caucasian participants with high CD4 counts and long-term viral suppression. In such a specific population, the “return to health” effect is minimized, and the absence of African ethnicity or female sex (groups often identified as most prone to INSTI-metabolic effect [18]) may explain the lack of significant metabolic changes reported in more diverse cohorts.

### 4.2. Glycemic Parameters

Regarding glucose metabolism, our analysis found no association between INSTI use and insulin resistance (HOMA-IR). This is consistent with a 2023 meta-analysis of sixteen studies, which generally found no increased risk of type 2 diabetes mellitus (T2DM) associated with INSTI compared to PI or NNRTIs, with the notable exception of specific African populations [18]. This supports the hypothesis that in a Caucasian, metabolically stable population, INSTI might not exert a clinically significant diabetogenic effect as in heterogenous populations.

### 4.3. Lipid Profile

While no statistically significant differences were found in the overall lipid profile, a marginal trend was observed for higher TC in the DRV/TAF (PI-based) group compared to the DTG/TAF (INSTI-based) group. Recent reviews have associated PI with more significant impact on lipid metabolism than INSTI [19]. The lack of statistical significance in our study likely results from the modest sample size and the isolated direction of this trend (*p* = 0.075) must be interpreted with high caution. It should be treated as potential statistical noise and an exploratory observation rather than a basis for clinical decisions regarding the choice between PI and INSTI in patients with pre-existing dyslipidemia.

We observed a weak inverse correlation between total ART duration and non-HDL cholesterol (R = −0.22, *p* = 0.038), accompanied by a non-significant trend for triglycerides. It could be expected that the longer the time of living with HIV and receiving ART is, the greater disturbances should be, yet we consider that this reflects the clinical characteristics of long-term treated patients in our setting. Patients with the longest treatment history in our cohort (>20 years) represent a highly selected group under continuous medical surveillance. In this subset, early metabolic interventions—whether pharmacological or lifestyle-based—are more likely to have been successfully implemented over years of care. These lower non-HDL levels may demonstrate the effectiveness of long-term lipid management in people living with HIV rather than a direct pharmacological effect of ART duration itself.

### 4.4. Study Context

We need to underline that our findings must be interpreted within the specific context of our cohort. We analyzed a strictly selected group of well-managed PLWH with consistent viral suppression. By intentionally excluding individuals with HBV/HCV co-infection and heavy alcohol use, we minimized major confounders often present in large heterogenous studies. This approach enhanced our internal validity and likely explains the lack of statistically significant differences between the evaluated ART regimens.

We consider that weight gain and metabolic disturbances in a clinically stable, well-treated population are shaped less by ART regimen and more by non-HIV factors, including dietary habits, physical activity levels, and psychosocial well-being [20]. Thus, the lack of significant findings in our study offers a relevant clinical perspective: in uncomplicated, baseline-healthy patients, modern ART appears metabolically safe, and the medication itself does not override standard metabolic risk factors.

Other studies with larger, more diverse cohorts include patients with a wider range of health conditions, where the specific metabolic effects of ART may be more pronounced.

Possibly, extending the observation period and/or including patients with longer exposure to specific treatment regimens (e.g., >10 years) could reveal more subtle distinctions between therapies. While many studies provide data on mid-term cardiometabolic complications, refs. [6,21], data assessing treatment duration as an independent risk factor is scarce.

### 4.5. Selection Bias and Generalizability

The recruitment process during the COVID-19 pandemic in 2021 introduced an unavoidable selection bias. Out of 392 eligible patients, only 93 (23.7%) were enrolled. During this period, the clinic operated primarily via teleconsultations. In-person visits were largely restricted to patients whose routine, mandatory laboratory monitoring fell within our recruitment window, while others were managed remotely with e-prescriptions. Consequently, demographic and clinical data for the 299 non-enrolled patients were not extracted for this study and cannot be retrospectively recovered. However, their key baseline characteristics can be inferred. These non-enrolled individuals passed the same screening phase to be included in the initial pool of 392 eligible patients (aged 18–65, virologically stable on ART for over two years, and free of HBV/HCV co-infections or severe liver diseases). Also, their successful management via teleconsultations suggests they were clinically stable, without acute or severe complaints requiring physical examination. Thus, the primary distinction between the enrolled and non-enrolled groups was likely administrative (scheduled routine blood work falling outside the recruitment window) rather than a fundamental difference in their underlying HIV or metabolic status.

However, our findings apply strictly to this specific urban, Caucasian, male-predominant, and coinfection-free cohort and should not be generalized to broader HIV populations.

### 4.6. Limitations

We acknowledge several other limitations of our study, primarily the cross-sectional design and modest sample size (*n* = 93). A major limitation of this study is the lack of a priori sample size justification and the small subgroup sizes (50 in the INSTI/TAF group vs. 28 in the PI/TAF group). These factors limit the statistical power of the analysis, meaning the study is likely underpowered to detect small but clinically relevant differences. Thus, the lack of significant differences between the regimens should be interpreted cautiously, as it might reflect a Type II error and not true clinical equivalence. Also, the relatively short median of ART duration (6.24 years) also limits the interpretation of long-term ART effects. A longer follow-up or a larger cohort with prolonged exposure to specific regimens could reveal some differences between therapies. Additionally, the use of a self-reported, non-validated questionnaire to assess lifestyle factors introduces a risk of recall and measurement bias. Additionally, the comparative analysis between ART classes is susceptible to confounding by indication. Individuals receiving boosted PIs may systematically differ from those on INSTI-based therapies regarding their past treatment histories, including previous virological failures, resistance profiles, or drug intolerances. This difference is partially reflected in our cohort by the significantly longer duration of the current regimen observed in the PI/TAF group. Due to the cross-sectional nature of the data, causal inferences regarding the metabolic effects of specific ARV classes cannot be made.

## 5. Conclusions

In our highly selected cohort of well-controlled people living with HIV, we observed no statistically significant associations between HIV-related factors (namely, ART regimen, treatment duration, and nadir CD4 count) and the presence of metabolic complications. Importantly, the limited sample size and lack of a priori power calculation mean our study was not powered to detect modest but clinically relevant differences. Therefore, the absence of statistical significance should not be interpreted as evidence of clinical equivalence between the studied regimens. These findings suggest that in a highly selected population, free from major metabolic confounders like chronic liver disease or viral hepatitis, the choice of a specific modern ARV agent may have a less pronounced impact on metabolic health than traditional risk factors. The single-center nature of our study and the risk of a Type II error must be considered when interpreting these results. Larger, similarly controlled studies are needed to further distinguish the metabolic effects of ARV agents from non-ART-related factors in stable patients living with HIV.

## Figures and Tables

**Table 1:** Demographic, clinical and virological characteristics across ART regimens.

Parameter	Total (*n* = 93)	INSTI/TAF (*n * = 50)	PI/TAF (*n* = 28)	*p*-Value	Effect Size r/OR	Test	ELV/TAF (*n* = 28)	DTG/TAF (*n* = 15)	DRV/TAF (*n* = 28)	*p*-Value	Effect Size η^2^/V	Test
Age (years)	39 (34–48)	38 (34–49)	42 (36–48)	0.25	r = 0.13	MW	35.5 (34–49)	43 (35–54)	41.5 (36–48)	0.087	η^2^ = 0.070	KW
Men (n)	77	42	23	0.94	OR = 1.14	χ^2^	24	11	23	0.6	V = 0.12	χ^2^
BMI (kg/m^2^)	24 (23–27.2)	24 (23–27)	26 (22–28)	0.65	r = 0.05	MW	23.9 (22.9–27.0)	24.8 (23.3–25.4)	25.7 (22–27.6)	0.8	η^2^ = 0.006	KW
Metabolic syndrome (n)	10	6	3	0.91	OR = 1.14 (95% CI: 0.26–4.94)	χ^2^	5	0	3	0.21	V = 0.21	χ^2^
Smokers (n)	27	15	9	0.84	OR = 0.90 (95% CI: 0.33–2.45)	χ^2^	8	5	9	0.94	V = 0.04	χ^2^
Alcohol intake (standard units)	0 (0–5.4)	0.0 (0–5.2)	1.8 (0–9.3)	0.33	r = 0.11	MW	1.35 (0–5.4)	0	1.8 (0–9.3)	0.18	η^2^ = 0.049	KW
Physical effort (units)	3.5 (2–6)	4.8 (3–7)	3.5 (2.5–5)	0.32	r = 0.11	MW	5.0 (2.5–7.5)	5.5 (2–15)	3.5 (2.5–5)	0.36	η^2^ = 0.029	KW
Lifetime weight amplitude (kg)	16 (9–25)	16 (10–25)	20 (9–25)	0.66	r = 0.05	MW	17 (9–25)	12 (4–30)	20 (9–25)	0.71	η^2^ = 0.010	KW
Viremia 20–200 (copies/mL)	14	6	7	0.27	OR = 0.41	χ^2^	2	1	7	0.2	V = 0.21	χ^2^
Viremia > 200 (copies/mL)	1	1	0	0.27	OR = 1.73	χ^2^	1	0	0	0.2	V = 0.21	χ^2^
Nadir CD4 (cells/μL)	(257–498)	374 (279–497)	338 (228–527)	0.64	r = 0.05	MW	370 (254–484)	450 (335–588)	338 (228–527)	0.41	η^2^ = 0.025	KW
Current CD4 (cells/μL)	611 (500–813)	625 (500–831)	612 (516–808)	0.71	r = 0.04	MW	658 (575–824)	591 (398–794)	612 (516–808)	0.44	η^2^ = 0.023	KW
Time on ART (years)	6.2 (4.6–8.5)	5.6 (3.3–4.2)	6.3 (5.0–9.8)	0.11	r = 0.18	MW	6.4 (4.4–8.0)	4.7 (4.2–7.7)	6.3 (5.0–9.8)	0.29	η^2^ = 0.035	KW
Time on current regimen (years)	4.1 (3.5–4.3)	4.0 (3.9–4.5)	4.3 (3.6–4.5)	**0.019**	r = 0.27	MW	3.9 (3.3–4.1)	4.2 (3.9–4.5)	4.3 (3.6–4.5)	**0.0052**	η^2^ = 0.150	KW

Continuous variables are presented as median (Q1–Q3) and analyzed using Mann–Whitney (MW) for two-group comparisons or Kruskal–Wallis (KW) for three-group comparisons (ELV/TAF, DTG/TAF, DRV/TAF). Categorical variables show the number of participants with the characteristic and were analyzed using Chi-square tests (χ^2^). The rank-biserial correlation (r) was used for continuous variables compared between two groups (Mann–Whitney U test), and eta-squared (η2) for multiple groups (Kruskal–Wallis test). For categorical variables, the Odds Ratio (OR) was calculated for two-group comparisons, and Cramer’s V was used for multiple-group comparisons. Abbreviations: TAF—tenofovir alafenamide; INSTI—integrase strand transfer inhibitor; PI—protease inhibitor; ELV—elvitegravir; DTG—dolutegravir; DRV—darunavir; CD4—cluster of differentiation 4 (current or nadir); TC—total cholesterol; LDL-C—low-density lipoprotein cholesterol; HDL-C—high-density lipoprotein cholesterol; non-HDL-C—non-high-density lipoprotein cholesterol; TG—triglycerides; HbA1c—glycated hemoglobin; HOMA-IR—homeostatic model assessment of insulin resistance; T2DM—type 2 diabetes mellitus; Q1—first quartile; Q3—third quartile.

**Table 2:** Metabolic characteristics across ART regimens.

Parameter	Total (*n* = 93)	INSTI/TAF (*n * = 50)	PI/TAF (*n* = 28)	*p*-Value	Effect Size r/OR	Test	ELV/TAF (*n* = 28)	DTG/TAF (*n* = 15)	DRV/TAF (*n* = 28)	*p*-Value	Effect Size η^2^/V	Test
TC (mg/dL)	193 (157–217)	185 (154–215)	206 (163–235)	0.16	r = 0.16	MW	195 (171–215)	166 (145–186)	206 (163–235)	0.075	η^2^ = 0.074	KW
LDL-C (mg/dL)	126 (91–145)	124 (92–141)	132 (91–159)	0.47	r = 0.08	MW	129 (106–150)	96 (81–130)	132 (91–159)	0.12	η^2^ = 0.061	KW
HDL-C (mg/dL)	50 (37–61)	47.6 (36.5–57.2)	53.4 (43–62.5)	0.26	r = 0.13	MW	47.6 (36.3–56.1)	44.3 (37–63.3)	53.4 (43–62.5)	0.38	η^2^ = 0.028	KW
non-HDL-C (mg/dL)	147 (108–165)	142 (106–162)	132 (101–177)	0.24	r = 0.13	MW	151 (120–163)	121 (96–147)	152 (111–177)	0.12	η^2^ = 0.061	KW
Triglycerides (mg/dL)	120 (89–166)	119 (78–163)	141 (100–207)	0.26	r = 0.13	MW	114 (96–171)	121 (75–155)	141 (100–207)	0.46	η^2^ = 0.022	KW
Dyslipidemia (n)	60	30	20	0.31	OR = 0.60 (95% CI: 0.22–1.62)	χ^2^	18	6	20	0.12	V = 0.24	χ^2^
Glucose (mg/dL)	92 (87–100)	93 (88–100)	93 (86–101)	0.83	r = 0.02	MW	93 (84–100)	90 (87–95)	93 (86–101)	0.63	η^2^ = 0.013	KW
HbA1c (%)	5.2 (4.9–5.4)	5.1 (4.9–5.5)	5.2 (5.0–5.3)	0.76	r = 0.03	MW	5.1 (5.0–5.4)	5.3 (4.8–5.6)	5.2 (5.0–5.3)	0.69	η^2^ = 0.011	KW
HOMA-IR	2.5 (1.6–4.1)	2.5 (1.6–4.2)	2.1 (1.5–4.3)	0.63	r = 0.05	MW	2.4 (1.5–4.1)	2.35 (1.3–3.0)	2.1 (1.5–4.3)	0.91	η^2^ = 0.003	KW
T2DM (n)	3	3	0	0.19	OR = 4.20	χ^2^	2	1	0	0.36	V = 0.17	χ^2^

Continuous variables are presented as median (Q1–Q3) and analyzed using Mann–Whitney (MW) for two-group comparisons or Kruskal–Wallis (KW) for three-group comparisons (ELV/TAF, DTG/TAF, DRV/TAF). Categorical variables show the number of participants with the characteristic and were analyzed using Chi-square tests (χ^2^). The rank-biserial correlation (r) was used for continuous variables compared between two groups (Mann–Whitney U test), and eta-squared (η2) for multiple groups (Kruskal–Wallis test). For categorical variables, the Odds Ratio (OR) was calculated for two-group comparisons, and Cramer’s V was used for multiple-group comparisons. Abbreviations: TAF—tenofovir alafenamide; INSTI—integrase strand transfer inhibitor; PI—protease inhibitor; ELV—elvitegravir; DTG—dolutegravir; DRV—darunavir; CD4—cluster of differentiation 4 (current or nadir); TC—total cholesterol; LDL-C—low-density lipoprotein cholesterol; HDL-C—high-density lipoprotein cholesterol; non-HDL-C—non-high-density lipoprotein cholesterol; TG—triglycerides; HbA1c—glycated hemoglobin; HOMA-IR—homeostatic model assessment of insulin resistance; T2DM—type 2 diabetes mellitus; Q1—first quartile; Q3—third quartile.

**Table 3:** Impact of nadir CD4 count and time of ART.

Parameter	Nadir CD4 Count		Time of ART	
	R	*p*-Value	R	*p*-Value
BMI	−0.06	0.56	0.15	0.16
TC	0	0.96	−0.16	0.13
LDL-C	0.06	0.60	−0.09	0.40
HDL-C	0.06	0.60	0.17	0.10
Non-HDL-C	0.03	0.80	−0.22	**0.038**
TG	0.03	0.78	−0.19	0.072
HbA1c	−0.07	0.50	0.13	0.21
HOMA-IR	−0.19	0.063	0.1	0.33

The *p*-values and R values refer to Spearman’s correlation coefficient. Abbreviations: CD4—cluster of differentiation 4; ART—antiretroviral therapy; TC—total cholesterol; LDL-C—low-density lipoprotein cholesterol; HDL-C—high-density lipoprotein cholesterol; non-HDL-C—non-high-density lipoprotein cholesterol; TG—triglycerides; HbA1c—glycated hemoglobin; HOMA-IR—homeostatic model assessment of insulin resistance.

**Table 4:** Impact of time of treatment with current regimen.

Parameter	All Regimens		Only InSTI or TAF	
	R	*p*-Value	R	*p*-Value
BMI	0.03	0.80	0.02	0.89
TC	−0.16	0.13	−0.16	0.14
LDL-C	−0.17	0.11	−0.18	0.098
HDL-C	0.09	0.40	0.11	0.30
Non-HDL-C	−0.13	0.20	−0.15	0.17
TG	−0.05	0.63	0.01	0.95
HbA1c	0.09	0.40	0.07	0.52
HOMA-IR	−0.1	0.35	−0.15	0.16

The *p*-values and R values refer to Spearman’s correlation coefficient. Abbreviations: CD4—cluster of differentiation 4; TC—total cholesterol; LDL-C—low-density lipoprotein cholesterol; HDL-C—high-density lipoprotein cholesterol; non-HDL-C—non-high-density lipoprotein cholesterol; TG—triglycerides; HbA1c—glycated hemoglobin; HOMA-IR—homeostatic model assessment of insulin resistance.

## Data Availability

The data supporting this research are not publicly available as they contain information that could compromise the privacy of research participants. However, anonymized data may be made available from the corresponding author upon reasonable request and with permission from the Ethics Committee of Wroclaw Medical University.
